# Surgery for colorectal cancer in people aged 80 years or older – complications, risks, and outcomes

**DOI:** 10.1097/MD.0000000000040696

**Published:** 2024-12-13

**Authors:** Jeong Hee Han, Byoung Chul Lee, Min Ju Kim, Jung Bum Choi, Hyuk Jae Jung, Hong Jae Jo

**Affiliations:** a Department of Surgery, Pusan National University Hospital, Busan, Republic of Korea; b Department of Surgery, Biomedical Research Institute, Pusan National University, School of Medicine, Busan, Republic of Korea; c Department of Surgery, Pusan National University, School of Medicine, Busan, Republic of Korea.

## Abstract

The number of older adult patients with colorectal cancer (CRC) is steadily increasing with the increasing aging population. However, healthcare professionals continue to approach treatment in older adult patients while considering the potential coexistence of complications relative to their age. There is a tendency to define and limit treatment options for managing “older adult patients” at relatively younger ages. Given the progression of aging societies and aging of patients with CRC, the impact of age on post-surgical outcomes should be analyzed to guide treatment decisions and ensure the highest quality of care for this population. This study aimed to compare outcomes in patients aged approximately 80 years who have undergone surgery after being diagnosed with CRC at the National Pusan University Hospital. This retrospective observational study included 502 patients who underwent surgery after being diagnosed with CRC at Pusan National University Hospital from January 2018 to December 2022. All surgeries were performed by a single surgeon. Older adult patients underwent open surgery more frequently. No significant differences in surgical outcomes or hospital stay were found between the two groups. Moreover, no notable differences were observed in overall complications, including major surgery-related complications such as anastomotic leakage, bleeding, and infection, between the two groups. However, pneumonia was significantly more common in the older patient group (*P* = .016). Among patients requiring emergency surgery, the older adult group demonstrated a significantly higher proportion of emergency surgeries and complications associated with regular surgeries compared with the younger group. In older adult patients, the risk of postoperative complications should not be determined solely based on age; a comprehensive assessment is necessary. However, in the case of emergency surgery, older adult patients may be relatively vulnerable compared with younger patients.

## 1. Introduction

The world’s population is aging rapidly, and this shift is closely linked to the rising incidence of many diseases, including colorectal cancer (CRC). In 2019, the global population aged 80 years or older was approximately 143 million. However, this number is expected to triple to 426 million by 2050.^[1]^ This demographic change has also influenced the incidence of CRC, which is becoming a significant health issue among older adults. In the United States, patients aged 70 and older account for 60% of those newly diagnosed with CRC; approximately two-thirds of CRC-related deaths occur in this age group.^[2]^ An analysis of data from 1980 to 2010 showed that CRC mortality rates increased with age, with men and women over 65 years experiencing a dramatic rise – 7.6 times and 5.7 times higher, respectively. This highlights the growing importance of addressing CRC in older populations.^[3]^ Korea is also part of the global aging trend. Busan, where our hospital is located, is one of the fastest-aging regions in the country. As of 2020, 4% of Busan’s population was aged 80 years and above, and this percentage is expected to double to 8% within the next decade, reaching 20% by 2050.^[3]^ With the growing proportion of older patients, there is an urgent need for in-depth research on the management and treatment of CRC in this population.

Older patients often face various physiological challenges during the recovery process after CRC surgery. As people age, organ function declines, resilience decreases, and comorbidities become more prevalent, leading to an increased risk of postoperative complications.^[4]^ These factors can prolong recovery time and negatively impact surgical outcomes. Therefore, healthcare providers may tend to offer less aggressive treatments to older patients, which can limit treatment options compared with that in younger patients.^[5]^ Traditionally, age has been considered a major risk factor for postoperative complications and mortality; it has been deemed appropriate for older patients to receive less aggressive treatments due to their higher risk and lower life expectancy.^[6,7]^ However, recent studies have suggested that CRC surgery is feasible and safe even in patients aged over 80 years.^[8,9]^ These studies indicate that using age alone to determine surgical candidacy is not appropriate and highlight the need for a more detailed analysis of postoperative outcomes in older patients. Given the increasing number of older patients with CRC, the lack of specific research on their postoperative outcomes remains a significant research gap, which limits the development of personalized treatment strategies that account for individual patient characteristics.

Therefore, the primary objective of this study was to compare the postoperative outcomes of CRC surgery between patients aged 80 years or older and those younger than 80 years. By analyzing key outcome indicators such as postoperative complications and hospital stay, we aimed to provide evidence that surgical treatment could be also safe and feasible for managing older patients with CRC. Additionally, we assessed the impact of surgery type by comparing outcomes between emergency and elective surgeries, focusing on how the type of surgery could affect postoperative outcomes in older patients. This study explored whether patients aged 80 years and above would exhibit significant differences in outcomes compared with younger patients, and whether these differences remain statistically significant after adjusting for variables such as complications and surgery type. Furthermore, using theoretical frameworks such as the “cumulative disadvantage theory,” we examined the relationship between age and health disparities, emphasizing the need for a multifaceted approach to health issues in older adults, rather than simply focusing on age, and how this approach could influence surgical interventions.^[4]^ This research could propose new guidelines for preoperative assessment and postoperative management in older patients, contributing to the improvement in surgical care for this population. The study would demonstrate that even patients aged 80 years and above can safely recover after surgical treatment of CRC, while also discussing the differences between emergency and non-emergency surgeries in older adults, highlighting the importance for early diagnosis and primary screening tests. Through this, we expect to provide significant implications not only for clinical practice but also for healthcare policy.

## 2. Methods

This study was a retrospective observational study conducted at Busan National University Hospital. The research ran from January 2018 to December 2022. Patient records were meticulously reviewed to ensure the accuracy and precision of the extracted data. Moreover, all surgeries were performed by a single surgeon, thereby minimizing variability in surgical techniques and decision-making processes.

### 2.1. Patient population

This study included patients aged approximately 80 years who underwent colorectal resection surgery following a diagnosis of CRC at our institution during the study period. Patients with multiple metastases who received chemotherapy instead of surgery, those who underwent local excision, and those who underwent surgery at another hospital after diagnosis were excluded from the study. A total of 502 patients were identified through hospital records; their medical records were retrospectively reviewed. The data were anonymized by removing personal identifying information, and the minimal risk to participants led to the waiver of written informed consent.

The variables evaluated were age, sex, comorbidities, American Society of Anesthesiologists (ASA) score for surgical and anesthetic risk, hemoglobin and albumin levels (as a measure of the patient’s nutritional status), emergency surgery (i.e., within 48 hours), elective surgery, surgical approach, combined operations, and operative name (right vs left and rectal). In the case of synchronous cancer, the operative name was based on the more severe lesion, pathological stage (TNM system; Union for International Cancer Control), and residual tumor. The surgical results analyzed included the surgical procedure, mean hospital stay, re-intervention rate (≤30 days), postoperative morbidity, and mortality (≤30 days). Data were collected from electronic medical records, and missing data were handled as specified in the statistical analysis subsection.

### 2.2. Ethical considerations

The study was conducted in accordance with the ethical principles of the Declaration of Helsinki, and the protocol was approved by the Institutional Review Board of Committee of Pusan National University Medical Center (IRB No. 2308-030-130). In the collection of research data, such as patient medical records, personally identifiable information was removed and anonymized. Furthermore, all related documents were encrypted and stored in a locked research facility, ensuring that only the researchers could have access. Research-related records must be retained for three years following the conclusion of the study, and once the retention period has expired, the documents would be destroyed in accordance with Article 16 of the Enforcement Decree of the Personal Information Protection Act.

### 2.3. Statistical analysis

**Figure 1.**
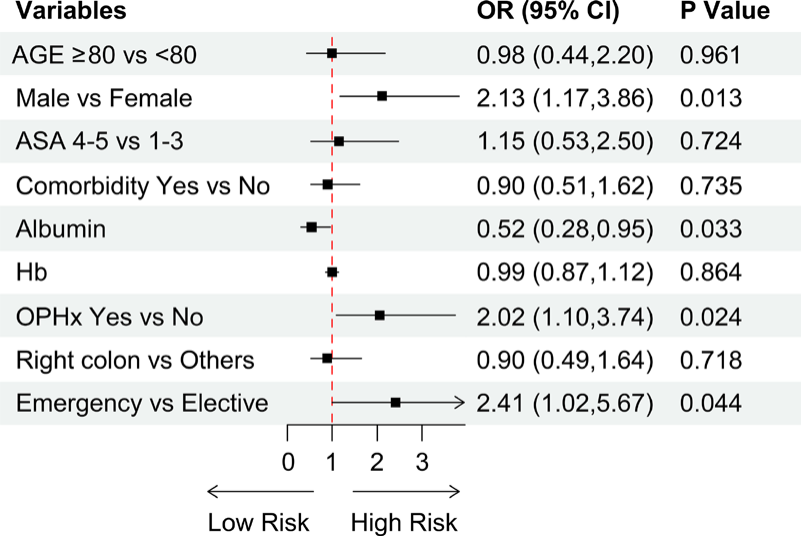
Adjusted odds ratio (OR) of complications (Overall). ASA = American Society of Anesthesiologists, Hb = hemoglobin, OPHx = operation history.

**Figure 2.**
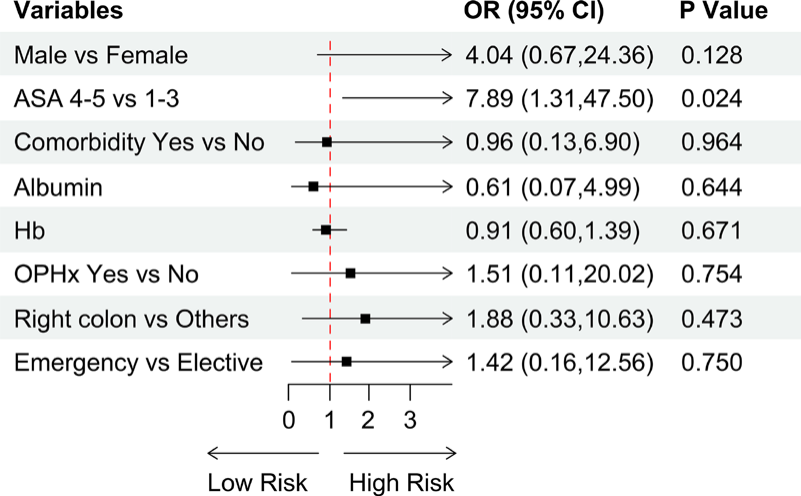
Adjusted odds ratio (OR) of complications (Subgroup: age ≥ 80 years). Hb = hemoglobin, OPHx = operation history.

Participant characteristics and group comparisons are presented in Tables 1–4. Continuous variables are expressed as means and standard deviations or medians and interquartile ranges based on the satisfaction of the normality assumption and the Shapiro–Wilk test. Categorical variables are presented as frequencies and percentages. The chi-squared or Fisher’s exact test was used for categorical variables, and the independent *t* test or Wilcoxon rank-sum test was used for continuous variables based on satisfaction of the normality assumption. To evaluate the factors affecting the response variables, postoperative complications and the length of hospital stay, univariate and multivariate analyses were conducted using generalized linear models. Logistic regression (binomial distribution with logit link) was used for the binary response variables (see Supplemental Digital Content 1, http://links.lww.com/MD/O65 and Supplemental Digital Content 2, http://links.lww.com/MD/O65 and Figs. 1 and 2), while a gamma distribution with a log link was applied for the continuous positive-valued variable. No imputation methods were applied for missing data. All statistical analyses were performed using R version 4.2.2 (R Core Team, 2022), with two-sided tests and a significance level set at 5%.

**Table 1:** Pre-surgical variables.

	Overall	Group A (<80 years)	Group B (≥80 years)	*P* value	SMD
n	502	439	63		
Age (median [IQR]), years	69.0 [61.0–75.0]	67.0 [60.0–73.0]	83.0 [81.0–85.0]	<. 001	2.529*
Sex (%)				.033	0.305
Male	297 (59.2)	268 (61.0)	29 (46.0)		
Female	205 (40.8)	171 (39.0)	34 (54.0)		
Albumin (g/dL) (median [IQR])	4.3 [4.3–4.6]	4.3 [4.0–4.6]	4.0 [3.6–4.2]	<.001	
Albumin (g/dL) [mean (SD)]	4.22 (0.51)	4.27 (0.50)	3.91 (0.45)		0.757
Hemoglobin (g/dL) (median [IQR])	12.4 [10.5–13.8]	10.4 [9.6–12.4]	12.2 [10.3–13.7]	<.001	
Hemoglobin (g/dL) [mean (SD)]	11.98 (2.35)	12.14 (2.33)	10.86 (2.23)		0.560
ASA Score (median [IQR])	2.0 [2.0–2.0]	2.0 [2.0–2.0]	2.0 [2.0–2.0]	.138	
ASA Score [mean (SD)]	2.07 (0.44)	2.06 (0.43)	2.14 (0.53)		0.177
ASA score (%)				.269	0.166
1–3	439 (87.6)	387 (88.4)	52 (82.5)		
4–5	62 (12.4)	51 (11.6)	11 (17.5)		
Comorbidity (%)					
Cancer history	54 (10.8)	46 (10.5)	8 (12.7)	.758	0.069
Hypertension	210 (41.9)	179 (40.9)	31 (49.2)	.264	0.168
Diabetes mellitus	121 (24.2)	109 (24.9)	12 (19.0)	.393	0.141
Liver cirrhosis	11 (2.2)	9 (2.1)	2 (3.2)	.637	0.070
Tuberculosis	10 (2.0)	8 (1.8)	2 (3.2)	.365	0.086
COPD	12 (2.4)	8 (1.8)	4 (6.3)	.052	0.230
Cerebrovascular accident	38 (7.6)	33 (7.6)	5 (7.9)	.804	0.014
Arrhythmia	26 (5.2)	22 (5.0)	4 (6.3)	.555	0.057
Angina/Myocardial infarction	39 (7.8)	34 (7.8)	5 (7.9)	>.99	0.006
Chronic kidney disease	19 (3.8)	18 (4.1)	1 (1.6)	.492	0.152
Previous abdominal surgery (%)	101 (20.2)	94 (21.4)	7 (11.3)	.091	0.276

ASA = American Society of Anesthesiologists score, COPD = chronic obstructive pulmonary disorder, IQR = interquartile, SMD = standardized mean difference.

*SMD > 0.8 is considered statistically significant.

**Table 2:** Surgical variables.

Characteristics	Overall	Group A (<80 years)	Group B (≥80 years)	*P* value	SMD
n	502	439	63		
Surgery				*.029	0.282
Elective surgery (%)	466 (93.0)	412 (94.1)	54 (85.7)		
Emergency surgery (<48 h) (%)	35 (7.0)	26 (5.9)	9 (14.3)		
Emergency surgery (<48 h) (%)				.739	0.418
Obstruction	26 (74.3)	20 (76.9)	6 (66.7)		
Perforation	8 (22.9)	5 (19.2)	3 (33.3)		
Bleeding	1 (2.9)	1 (3.8)	0 (0.0)		
Surgical approach (%)				.019	0.403
Laparoscopic	437 (87.2)	386 (88.1)	51 (81.0)		
Open	49 (9.8)	37 (8.4)	12 (19.0)		
Laparoscopic to Open	15 (3.0)	15 (3.4)	0 (0.0)		
Combined surgery (%)				.801	0.160
0	447 (89.2)	389 (88.8)	58 (92.1)		
1	50 (10.0)	45 (10.3)	5 (7.9)		
2	4 (0.8)	4 (0.9)	0 (0.0)		
Operation name (%)^[10]^				.044	0.283
Right colon	156 (31.1)	129 (29.4)	27 (42.9)		
Left colon/rectum	346 (68.9)	310 (70.6)	36 (57.1)		
Pathological stage (%)				.065	0.422
0	20 (4.0)	18 (4.2)	2 (3.2)		
1	109 (22.0)	103 (23.8)	6 (9.5)		
2	137 (27.6)	119 (27.5)	18 (28.6)		
3	171 (34.5)	142 (32.8)	29 (46.0)		
4	59 (11.9)	51 (11.8)	8 (12.7)		
Residual tumor (%)				.157	0.283
0	462 (92.2)	405 (92.5)	57 (90.5)		
1	11 (2.2)	11 (2.5)	0 (0.0)		
2	28 (5.6)	22 (5.0)	6 (9.5)		

* Significance level of 5% (two-tailed test);^[10]^: reference 44 in the manuscript

**Table 3:** Postoperative morbidity

Characteristic	Overall	Group A (<80 years)	Group B (≥80 years)	*P* value	SMD
n	502	439	63		
Hospitalization day (median [IQR])	7.0 [7.0–8.8]	7.0 [7.0–8.0]	7.0 [7.0–9.0]		
Hospitalization day (mean (SD))	9.71 (7.54)	9.78 (7.88)	9.17 (4.45)	.368	0.095
Postoperative mortality (%)				.235	0.144
No	500 (99.6)	438 (99.8)	62 (98.4)		
Yes	2 (0.4)	1 (0.2)	1 (1.6)		
Re-intervention (%)				.315	0.104
No	493 (98.2)	432 (98.4)	61 (96.8)		
Yes	9 (1.8)	7 (1.6)	2 (3.2)		
Complication (%)				>.99	0.018
No	433 (86.3)	379 (86.3)	54 (85.7)		
Yes	69 (13.7)	60 (13.7)	9 (14.3)		
Anastomotic site leak	13 (2.6)	11 (2.5)	2 (3.2)	.672	0.040
Intra-abdominal infection	11 (2.2)	9 (2.1)	2 (3.2)	.636	0.071
Surgical site infection	20 (4.0)	19 (4.3)	1 (1.6)	.493	0.162
Ileus	15 (3.0)	14 (3.2)	1 (1.6)	.706	0.105
Bleeding	0 (0.0)	0 (0.0)	0 (0.0)	NA	
Urinary tract infection	1 (0.2)	1 (0.2)	0 (0.0)	>.99	0.068
Pneumonia	5 (1.0)	2 (0.5)	3 (4.8)	*.016	0.273
Acute kidney injury	0 (0.0)	0 (0.0)	0 (0.0)	NA	
Myocardial infarction	0 (0.0)	0 (0.0)	0 (0.0)	NA	
Stroke	0 (0.0)	0 (0.0)	0 (0.0)	NA	
Sepsis	0 (0.0)	0 (0.0)	0 (0.0)	NA	

IQR = interquartile range, NA = not applicable, SD = standard deviation, SMD = standardized mean difference.

* Significance level of 5% (two-tailed tests).

**Table 4:** Older adult patients

Patients ≥ 80 years of age					
		Emergency			
Characteristic	Overall	Elective	Emergency	*P* value	SMD
n	63	54	9		
Operation (%)				.480	0.300
Right colon	27 (42.9)	22 (40.7)	5 (55.6)		
Left colon/rectum	36 (57.1)	32 (59.3)	4 (44.4)		
Hospitalization day (median [IQR])	7.0 [7.0–9.0]	7.0 [7.0–8.0]	9.0 [7.0–9.0]	.271	
Hospitalization day (mean (SD))	9.17(4.45)	9.19 (4.62)	9.11 (3.48)	.018	0.018
Postoperative mortality (%)				>.99	0.194
No	62 (98.4)	53 (98.1)	9 (100.0)		
Yes	1 (1.6)	1 (1.9)	0 (0.0)		
Re-intervention (%)				>.99	0.277
No	61 (96.8)	52 (96.3)	9 (100.0)		
Yes	2 (3.2)	2 (3.7)	0 (0.0)		
Complications (%)				.604	0.245
No	54 (85.7)	47 (87.0)	7 (77.8)		
Yes	9 (14.3)	7 (13.0)	2 (22.2)		
Anastomotic site leak	2 (3.2)	2 (3.7)	0 (0.0)	>.99	0.277
Intra-abdominal infection	2 (3.2)	2 (3.7)	0 (0.0)	>.99	0.277
Surgical site infection	1 (1.6)	1 (1.9)	0 (0.0)	>.99	0.194
Ileus	1 (1.6)	1 (11.1)	0 (0.0)	.143	0.500
Bleeding	0 (0.0)	0 (0.0)	0 (0.0)	NA	
Urinary tract infection	0 (0.0)	0 (0.0)	0 (0.0)	NA	
Pneumonia	3 (4.8)	1 (1.9)	2 (22.2)	.051	0.659
Acute kidney injury	0 (0.0)	0 (0.0)	0 (0.0)	NA	
Myocardial infarction	0 (0.0)	0 (0.0)	0 (0.0)	NA	
Stroke	0 (0.0)	0 (0.0)	0 (0.0)	NA	
Sepsis	0 (0.0)	0 (0.0)	0 (0.0)	NA	

IQR = interquartile range, NA = not applicable, SD = standard deviation, SMD = standardized mean difference.

## 3. Results

The mean age of the patients was 69.0 years, 297 (59.2%) were male individuals, and 63 (12.5%) were aged ≥ 80 years. Patients were categorized into the older adult group (≥80 years) and the younger adult group (<80 years).

### 3.1. Presurgical variables

The preoperative patient variables and the logistic regression results are shown in Table 1. No significant differences were found between the groups regarding ASA scores, comorbidities, or previous abdominal surgery. The hemoglobin and albumin levels were observed to be higher in the older adult group. Regarding sex, there were more male patients in the younger adult group and more female individuals in the older adult group.

### 3.2. Surgical variables

The older adult group exhibited a significant increase in the number of emergency surgeries, and open surgeries were more frequent (Table 2). Moreover, right-sided colon cancer was more prevalent in older adult patients. However, there were no significant differences in pathological stages or residual tumors.

### 3.3. Postoperative morbidity

There was no significant difference between the two groups regarding postoperative complications or length of hospital stay (Table 3). However, pneumonia was more prevalent in the older adult group. A multivariable logistic regression analysis was performed, and the results are shown in Supplemental Digital Content 1, http://links.lww.com/MD/O65 and Figure 1. Significant risk factors included male sex (Odds ratio [OR] 2.13, *P = *.013), previous surgery history (OR 2.02, *P = *.024), low albumin levels (OR 0.52, *P = *.033), and emergency surgery (OR 2.41, *P = *.044).

### 3.4. Older adult patients

The rate of emergency surgery was higher in the older adult group (Table 2). A multivariable logistic regression analysis for patients in the older adult group is shown in Supplemental Digital Content 2, http://links.lww.com/MD/O65 and Figure 2. The analysis identified a significantly higher risk for patients with an ASA score of 4 to 5 compared to those with scores of 1 to 3 (OR 7.89, *P = *.024). Although there was no significant difference in complications between emergency and scheduled surgeries (Table 4), the crude OR was 1.9, indicating an approximately 2-fold increase in risk.

## 4. Discussion

In this study, we analyzed surgical outcomes in patients aged approximately 80 years. In line with our hypothesis, the results showed no significant differences in the overall complication rates between both groups. Although the sample size in the older adult group was relatively smaller than that in the younger adult group (63 vs 439), which may have affected the observed frequency of complications, our findings may be reliable as age was not significantly different between the groups. However, a significant difference was observed in the incidence of pneumonia, which is consistent with previous studies suggesting that age may be a risk factor for pneumonia.^[11,12]^ At our hospital, to prevent pneumonia complications in older patients, particularly those at higher risk (such as those aged ≥ 65 years, those with lung or heart disease, or an Eastern Cooperative Oncology Group performance status of ≥ 2), we provide preoperative patient education focusing on respiratory rehabilitation. Additionally, upon admission, we consult with the rehabilitation department to initiate respiratory and physical rehabilitation.^[13]^ The multivariable logistic regression analysis revealed that in the overall group, male sex, albumin levels, and emergency surgery were associated with complications, while in the elderly group, complications were linked to ASA scores of 4 to 5. These findings show a similar trend with previous studies.^[14,15]^

Under these circumstances, considering age alone as a risk factor for surgery may not be warranted. The complexity of cancer management in older adults is due to individual variations in health status, activity levels, age-related physiological changes, and differences in life expectancies. Moreover, patients aged ≥ 80 years are often underrepresented in randomized clinical trials, leading to a lack of systematic knowledge on the benefit-risk ratio of treatment options in this age group.^[16]^ Traditionally, many researchers have defined older adult patients using relatively younger ages, possibly resulting in uneven treatment practices in this population.^[6]^ Some past studies have reported higher postoperative complications and mortality rates in older adult patients with CRC, suggesting lower cancer survival rates in this age group.^[6,7]^ However, the high mortality rate in older adult patients with CRC can be attributed to excess mortality in the first month after diagnosis, suboptimal treatment, high postoperative mortality, and inadequate risk stratification.^[17,18]^ Moreover, some studies failed to demonstrate a significant association between age and overall survival using multivariate analysis.^[19,20]^ Recent studies have indicated that surgical treatment of CRC in older adult patients is safe and comparable to that in younger age groups regarding postoperative complications.^[21,22]^ In our study, no significant associations were observed between age and comorbidities.

This trend cannot be explained simply by generational differences. Comparing the overall health status of the current and previous generations is challenging, and there is insufficient evidence to support the notion that today’s older adult population is healthier than that of previous generations.^[23]^ However, advancements in the treatment of comorbid conditions; pre- and postoperative managements, and minimally invasive surgical techniques have led to lower surgical thresholds and better postoperative outcomes.^[24–26]^ As evident from the findings of this study, using age as the sole criterion for treatment decisions, particularly in older patients, may not be appropriate. Although aging and comorbidities may be associated, being older does not necessarily mean having more comorbidities because individual differences in comorbidities can significantly vary. Moreover, the exact roles of comorbidities as prognostic factors have not been clarified. Studies have demonstrated that comorbidities in older adult patients with head and neck cancers are associated with poor surgical outcomes when considering overall survival.^[27–30]^ However, population-based studies have reported negligible effects on the overall prognosis in patients with cancer.^[31,32]^ Similarly, no significant association was observed between comorbidities, postoperative complications, and short-term mortality rates. Given the unclear relationship between comorbidities and postoperative outcomes in older adult patients with cancer, treatment decisions should not be solely based on the presence or number of comorbidities.

On the other hand, in older adult patients, emergency surgeries can pose a higher risk compared with elective procedures.^[33–35]^ Situations requiring emergency surgery typically involve more advanced disease progression and higher invasiveness compared with elective procedures. Moreover, older adult patients often have reduced physiological reserves to cope with stressors, further increasing the risk associated with emergency surgeries.^[36,37]^ Although this study did not yield statistically significant results due to an insufficient sample size, it was observed that the relative risk of complications was higher in older adult patients undergoing emergency surgery (OR 1.9). Emergency surgeries are more common in older adult patients, as explained by the higher likelihood of living alone and lower awareness of early cancer symptoms. Older adult patients often have inadequate awareness of diseases and a lack of understanding of the recommended screening guidelines and their importance. This trend becomes more pronounced with increasing age, underscoring the importance for patient education and appropriate screening in primary care to prevent emergency surgery in older adult patients.^[5,38]^ In Korea, the primary screening test for CRC is the fecal immunochemical test, which is conducted for individuals aged 50 years and older. If the test result is positive, a colonoscopy is performed. Although the fecal immunochemical test is less expensive and noninvasive compared with a colonoscopy, it has lower sensitivity and is unable to detect precancerous lesions. Currently, the National Cancer Center is conducting a program to implement colonoscopy as the primary screening method, with the results expected to be published in 2025. Additionally, many countries, including Korea, limit CRC screening in individuals over the age of 80 years. Considering the impact of emergency surgeries on older patients with CRC and the relatively low cost of colonoscopy in Korea (ranging between 76 and 115 USD), revisions to colonoscopy guidelines and screening recommendations may be warranted.

Functional measures are better than chronological ages in predicting survival and tolerance to cancer treatment. Although patient’s chronological age is based on time alone, their physiological age reflects the cumulative effect of medical and psychosocial stressors (e.g., caregiving or loss of independence) on the aging process, which can affect life expectancy.^[39]^ In this context, the “cumulative disadvantage theory” suggests that accumulated comorbidities, psychological stress, and physical changes over time can significantly impact a patient’s functional status and postoperative outcomes. Therefore, a multifaceted approach based on the patient’s health status and functional indicators, rather than age alone, is necessary.^[4]^ The best estimate of an individual’s physiological age can be obtained via functional measures, and individual functional measures can be conducted through a geriatric assessment.^[40]^ For this reason, the National Comprehensive Cancer Network and the International Society of Geriatric Oncology recommend geriatric assessments in the clinical setting for older adult patients with cancer. However, the efficacy of geriatric assessments has not been fully established, and randomized controlled trials have not been conducted.^[41]^ Ultimately, although included in the guidelines, geriatric assessments are currently mainly utilized to facilitate a multidimensional understanding of the patient rather than being fully employed to determine personalized treatment options. Further research is needed to validate the use of geriatric assessments and their potential to improve tailored medical care and oncological outcomes in older adult patients.

This study has some limitations. First, as it is a retrospective observational study, there is a possibility of bias related to patient characteristics, which could affect the outcomes. In particular, older patients who are relatively healthier may tend to opt for surgical treatment, which might explain why no significant differences in complications were observed. Second, the process of data collection based on medical records could lead to missing or inaccurate information. Third, the small sample size of the older patient group (63 patients) may have also affected the statistical significance of the results. Fourth, the small number of patients who underwent emergency surgery (54 patients vs 9 patients) made it difficult to accurately evaluate the rate of complications between emergency and elective surgeries. This imbalance in sample size made achieving statistical significance challenging, limiting the ability to fully assess the true impact of emergency surgery on older patients.

Finally, the limited follow-up period in this study restricted our ability to evaluate long-term postoperative complications or survival rates. Since this study was conducted at a single institution, whether similar results would be observed in other regions or hospitals remains uncertain. To overcome these limitations, future prospective multi-center studies with larger sample sizes and longer follow-up periods are necessary. Such studies would allow for a clearer comparison of emergency and elective surgeries in older patients and provide a better understanding of long-term surgical outcomes. Furthermore, multivariate analyses that consider comorbidities and functional status are essential for developing preoperative optimization strategies tailored to older patients, ultimately improving postoperative outcomes in this population.

In conclusion, this study demonstrates that surgical treatment can be safely performed in older patients with CRC and emphasizes that treatment decisions should be based on individual health status and functional indicators rather than age alone. It highlights the importance of perioperative management to prevent pneumonia and suggests that such preventive strategies can be applied in other healthcare settings as well. Additionally, the development of preoperative optimization programs could contribute to improving postoperative outcomes in older patients. Although the small sample size limited the ability to draw definitive conclusions regarding emergency surgeries, the higher risks associated with emergency procedures in older patients should be considered. Future research should focus on prospective interventional studies to explore the impact of preoperative optimization programs, including nutritional support and rehabilitation exercises, on reducing postoperative complications. Furthermore, it will be important to conduct multi-center collaborations to ensure a sufficient patient population for a detailed analysis of the risks associated with emergency surgeries in older patients.

## Acknowledgments

We thank the Department of Biostatistics of the Biomedical Research Institute of the Pusan National University Hospital.

## Author contributions

**Supervision:** Min Ju Kim, Jung Bum Choi, Hyuk Jae Jung, Hong Jae Jo.

**Writing – original draft:** Jeong Hee Han.

**Writing – review & editing:** Byoung Chul Lee.

## Supplementary Material


