# Supplementary material for: Surgery for colorectal cancer in people aged 80 years or older – complications, risks, and outcomes
Source: Medicine (Baltimore). 2024 Dec 13;103(50):e40696. doi: 10.1097/MD.0000000000040696 (PMC11651447; doi:10.1097/MD.0000000000040696)
Supplement: Supplementary file 1 [file medi-103-e40696-s001.docx]

**Supplemental Digital Content 1. Crude and adjusted odds ratios from univariate and multivariate logistic regression models for risk of complication in the entire cohort**

| Variable | Levels | Complication | | Crude OR | *P*-value | Adjusted OR | | *P*-value |
| --- | --- | --- | --- | --- | --- | --- | --- | --- |
|  |  | No (n=433) | Yes (n=69) | (95% CI) |  | | (95% CI) |  |
| Sex (%) | Male | 249 (83.8) | 48 (16.2) | 1.689 (0.977–2.919) | .06 | | 2.127 (1.188–3.938) | *.013 |
|  | Female | 184 (89.8) | 21 (10.2) |  |  | |  |  |
| Age (%) | Group B (≥80 years) | 54 (85.7) | 9 (14.3) | 1.053 (0.494–2.243) | .894 | | 0.980 (0.414–2.114) | .961 |
|  | Group A (<80 years) | 379 (86.3) | 60 (13.7) |  |  | |  |  |
| ASA (%) | 4–5 | 51 (82.3) | 11 (17.7) | 1.445 (0.712–2.936) | .308 | | 1.150 (0.509–2.427) | .724 |
|  | 1–3 | 382 (87.0) | 57 (13.0) |  |  | |  |  |
| Comorbidity (%) | Yes | 264 (86.6) | 41 (13.4) | 0.937 (0.558–1.573) | .807 | | 0.905 (0.508–1.635) | .735 |
|  | No | 169 (85.8) | 28 (14.2) |  |  | |  |  |
| Albumin (g/dL) (mean (SD)) |  | 4.25 (0.50) | 4.04 (0.51) | 0.467 (0.292–0.748) | .001 | | 0.529 (0.288–0.978) | *.033 |
| Hb(g/dL) (mean (SD)) |  | 12.04 (2.36) | 11.58 (2.29) | 0.921 (0.829–1.025) | .128 | | 0.994 (0.873–1.137) | .864 |
| Previous abdominal surgery | Yes | 81 (80.2%) | 20 (19.8%) | 1.769 (0.980–3.101) | .051 | | 2.020 (1.103–3.743) | *.024 |
|  | No | 351 (87.8%) | 49 (12.3%) |  |  | |  |  |
| Operation | Right | 135 (86.5%) | 21 (13.5%) | 0.966 (0.547–1.657) | .901 | | 0.903 (0.492–1.643) | .718 |
|  | Left/Rectum | 298 (86.1%) | 48 (13.9%) |  |  | |  |  |
| Emergency surgery |  | 25 (71.4%) | 10 (28.6%) | 2.814 (1.234–6.004) | .010 | | 2.41 (1.02–5.67) | *.044 |

ASA, American Society of Anesthesiologists score; CI, confidence interval; SD, standard deviation; OR, odds ratio; Hb, hemoglobin

*: significance level of 5% (two-tailed tests)

**Supplemental Digital Content 2. Crude and adjusted odds ratios from univariate and multivariate logistic regression models for risk of complication in participants aged 80 years and older**

| Variable | Levels | Complication | | Crude OR | *P*-value | Adjusted OR | *P*-value |
| --- | --- | --- | --- | --- | --- | --- | --- |
|  |  | No (n=54) | Yes (n=9) | (95% CI) |  | (95% CI) |  |
| Sex (%) | Male | 23 (79.3%) | 6 (20.7%) | 2.696 (0.641–13.849 | .191 | 2.639 (0.544–15.599) | .242 |
|  | Female | 31 (91.2%) | 3 (8.8%) |  |  |  |  |
| ASA (%) | 4–5 | 6 (54.5%) | 5 (45.5%) | 10.000 (2.127–51.898) | .004* | 10.112 (1.861–65.612) | .009* |
|  | 1–3 | 48 (92.3%) | 4 (7.7%) |  |  |  |  |
| Comorbidity (%) | Yes | 36 (83.7%) | 7 (16.3%) | 1.750 (0.376–12.557) | .511 | 0.837 (0.124–6.888) | .854 |
|  | No | 18 (90.0%) | 2 (10.0%) |  |  |  |  |
| Albumin (g/dL) (mean (SD)) |  | 3.92 (0.48) | 3.83 (0.25) | 0.651 (0.138–3.183) | .584 | 0.599 (0.066–5.147) | .635 |
| Hb (g/dL) (mean (SD)) |  | 10.96 (2.15) | 10.27 (2.75) | 0.866 (0.615–1.194) | .386 | 0.924 (0.586–1.408) | .716 |
| Previous abdominal surgery | Yes | 6 (85.7%) | 1 (14.3%) | 0.979 (0.048–6.888) | .985 | 1.111 (0.025–14.63) | .943 |
|  | No | 47 (85.5%) | 8 (14.5%) |  |  |  |  |
| Operation | Right | 22 (81.5%) | 5 (18.5%) | 1.818 (0.435–8.08) | .410 | 1.589 (0.226–10.571) | .623 |
|  | Left/Rectum | 32 (88.9%) | 4 (11.1%) |  |  |  |  |
| Emergency surgery |  | 7 (77.8%) | 2 (22.2%) | 1.918 (0.252–10.103) | .468 | 1.191 (0.115–8.629) | .870 |

ASA, American Society of Anesthesiologists score; CI, confidence interval; SD, standard deviation; OR, odds ratio; Hb, hemoglobin

*: significance level of 5% (two-tailed tests)
